# Determining the development stage of the ossification centers around the elbow may aid in deciding whether to use ESIN or not in adolescents’ forearm shaft fractures

**DOI:** 10.1080/17453674.2021.1912895

**Published:** 2021-04-18

**Authors:** Markus Stöckell, Tytti Pokka, Nicolas Lutz, Juha-Jaakko Sinikumpu

**Affiliations:** a Department of Children and Adolescents, Oulu University Hospital; PEDEGO Research Group, Oulu Childhood Fracture and Sports Injury Study, Oulu University and Oulu University Hospital; Medical Research Council, Oulu University, Oulu, Finland;;; b Department of Pediatric Surgery, Lausanne University Hospital , Lausanne , Switzerland

## Abstract

Background and purpose — Elastic stable intramedullary nailing (ESIN) is the preferred method of operative stabilization of unstable pediatric forearm shaft fractures. However, the decision whether to use ESIN or open reduction and internal fixation (ORIF) in older children or teenagers is not always straightforward. We hypothesized that the development stage of the elbow would aid in evaluating the eligibility of the patient for ESIN.

Patients and methods — All eligible children, aged <16 years who were treated with ESIN in Oulu University Hospital, during 2010–2019 were included (N = 70). The development stages of 4 ossification centers were assessed according to the Sauvegrain and Diméglio scoring. The proportion of impaired union vs. union was analyzed according to bone maturity, by using the optimal cutoff-points determined with receiver operating characteristics (ROC).

Results — Development stage ≥ 6 in the olecranon was associated with impaired union in 20% of patients, compared with none in stages 1–5 (95% CI of difference 8% to 24%). Trochlear ossification center ≥ 4 was associated with impaired union in 17% of patients (CI of difference 7% to 36%) and lateral condyle ≥ 6 in 13% of patients (CI of difference 3.4% to 30%). Proximal radial head ≥ 5.5 was associated with impaired union in 18% of patients (CI of difference 7% to 39%).

Interpretation — Recognizing the rectangular or fused olecranon ossification center, referring to stage ≥ 6, was in particular associated with impaired fracture healing. This finding may aid clinicians to consider between ESIN and plating, when treating forearm shaft fracture of an older child or teenager.

Pediatric forearm shaft fractures comprise 6% of all childhood fractures. They occur most frequently in children aged 5–14 years (Wall 2016, Joeris et al. 2017, Alrashedan et al. 2018). Most can be treated nonoperatively, and this is particularly recommended in children < 9 years (Zionts et al. 2005, Franklin et al. 2012). Older children are more prone to complications such as nonunion and redisplacement (Asadollahi et al. 2017). Their longer fracture healing time and less pronounced remodeling capacity have resulted in a trend toward operative management recently (Sinikumpu et al. 2012).

Elastic stable intramedullary nailing (ESIN) is the preferred method to fix forearm shaft fractures in children. The method spares periosteal blood supply and surgical wounds are usually far from the fracture. ESIN produces good angular and longitudinal stability (Wall 2016). In older children and teenagers open reduction and internal fixation (ORIF) is optional (Herman and Marshall 2006). Their fractures are more prone to complications and even minor displacement may result in shortening and angulation, thus decreasing pro- and supination, similarly to adult patients (Rehman and Sokunbi 2010). However, the calendar age of a patient does not always match the maturation of the skeleton, making it challenging to select between pediatric-like or adult-like treatment.

Bone age of the patient would help the clinician when choosing between ESIN and plating in older children. Bone age could be assessed by additional radiographs of the hand or iliac spine. However, keeping in mind that there are several ossification centers in the elbow, which develop in a particular order in a growing child, we hypothesized that higher development stage of elbow ossification centers would be associated with impaired healing of forearm shaft fractures stabilized by ESIN. We aimed to find a method to predict impaired union of forearm shaft fractures treated by ESIN, by using the Sauvegrain classification system for bone age (Sauvegrain et al. 1962).

## Patients and methods

This is a population-based study that included all eligible consecutive patients, aged less than 16 years, who had been treated for a forearm shaft fracture using ESIN in the study center. The study center is the only full-time pediatric trauma unit in the geographical catchment area of Oulu region, in Northern Finland. The child population at risk in the study area is approximately 87,000 annually.

All primary and follow-up radiographs were reviewed to confirm inclusion; only diaphyseal both-bone fractures treated by titanium alloy elastic stable intramedullary nails were included and AO classification was used to subgroup the patients (Slongo et al. 2007). If more than 1 forearm shaft fracture was found in the same patient, the 1st of them was included for analysis. There were 2 such patients. No patient had a pathological fracture or bone dysplasia. Altogether 112 patients were primarily reviewed and finally 70 of these met the inclusion criteria. 36 patients were treated surgically by using Kirschner wires or other straight nails, intramedullary or other surgical procedures, and they were therefore excluded. 4 patients were excluded due to incorrect diagnosis and 2 more had an isolated fracture of the ulna.

Altogether 4 impaired unions were detected; 1 patient with nonunion had undergone an ossifying operation. 3 patients showed delayed union but ossified finally, after 6, 8, and 11 months of follow-up. Insufficient or lacking callus, fracture line visibility, or lacking cortical healing were assessed according to the Lane–Sandhu score (Bhandari et al. 2002). The patients who suffered from nonunion were immobilized for a mean 5.3 weeks, compared with 4.0 weeks among patients with expected fracture union. In the nonunion group, all patients had a nail–intramedullary canal ratio of at least 0.4, while 4.9% of cases in the union group were stabilized by using thinner nails with a nail–intramedullary canal ratio of < 0.4. There was no difference in the preferred orientation of the tips of the nails in the radius and ulna in comparison, demonstrating correct direction of the pre-bent nails, while none in the nonunion group and 16 patients in the union group achieved this in textbook fashion.

The patients were mean 9.8 years (2–15) of age. Half of them were boys (n = 36). 43 were classified as type 22-D/5.1, 14 were type 22-D/5.2, 11 were type 22-D/4.1, and 2 were type 22-D4.2. Trampoline jumping was the most frequent cause of injury (26): 23 from a fall < 1 m and 15 from more than 1 m. 12 children were treated surgically following failure of nonoperative treatment (loss of reduction).

**Figure 1.**
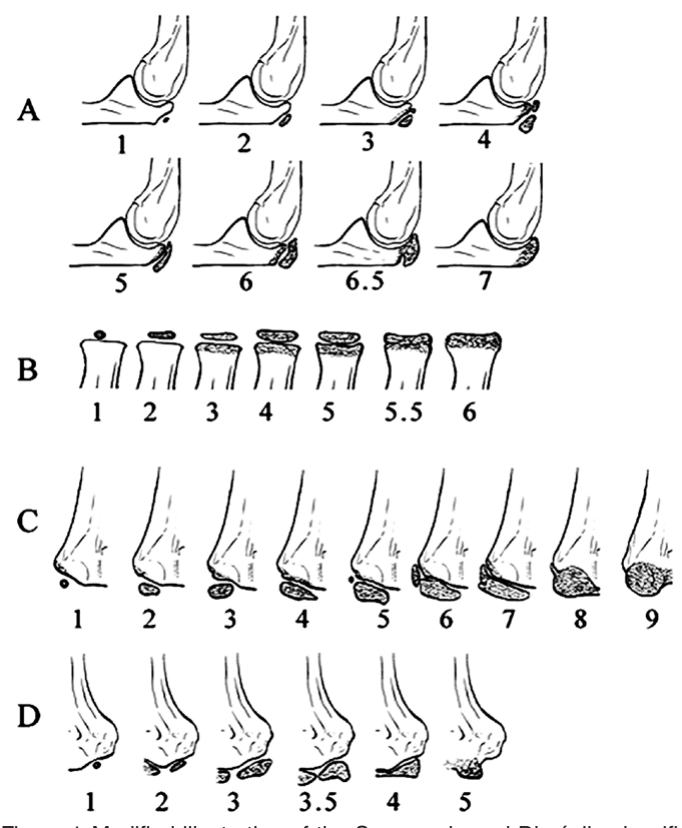
Modified illustration of the Sauvegrain and Dimйglio classification of maturation of the secondary ossification centers around the elbow. This staging was used in this research to find the optimal cutoff-point for (A) olecranon, (B) proximal radial head, (C) lateral condyle of the humerus, and (D) trochlea of the humerus.

**Figure 2.**
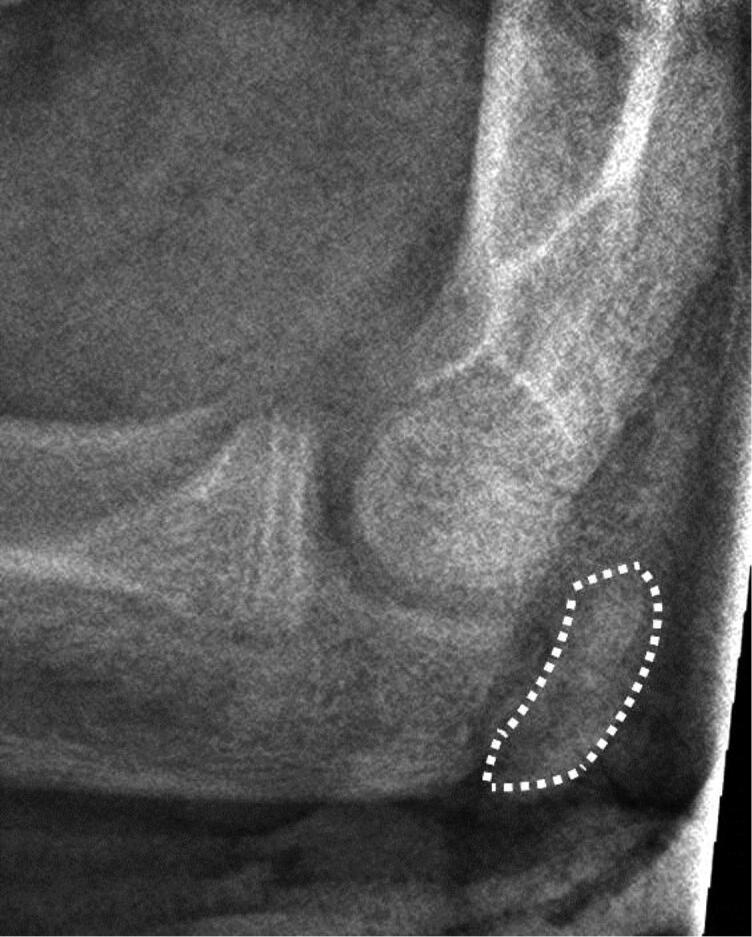
The elbow has been captured on the lateral view of the forearm radiograph and the secondary ossification center of the olecranon has been marked with a border (dotted line). The patient is a male aged 12. According to the Sauvegrain and Dimйglio method the olecranon ossification stage with a rectangular shape is 6. This stage was found to be a cutoff point in association with disturbed bone union after ESIN of forearm shaft fracture.

We examined the association between impaired union and the development status of the elbow ossification centers: olecranon, trochlea, lateral condyle, and proximal radius. The maturation stage, referring to bone age, was classified by using the Sauvegrain method, modified by Diméglio (Sauvegrain et al. 1962, Diméglio et al. 2005, Charles et al. 2007) (Figure 1). In case of uncertain selection between 2 consecutive classification groups, the patient was classified to the higher one. In the olecranon ossification center, rectangular apophysis demonstrated stage 6 in the lateral view of the radiographs (Figure 2).

**Figure 3.**
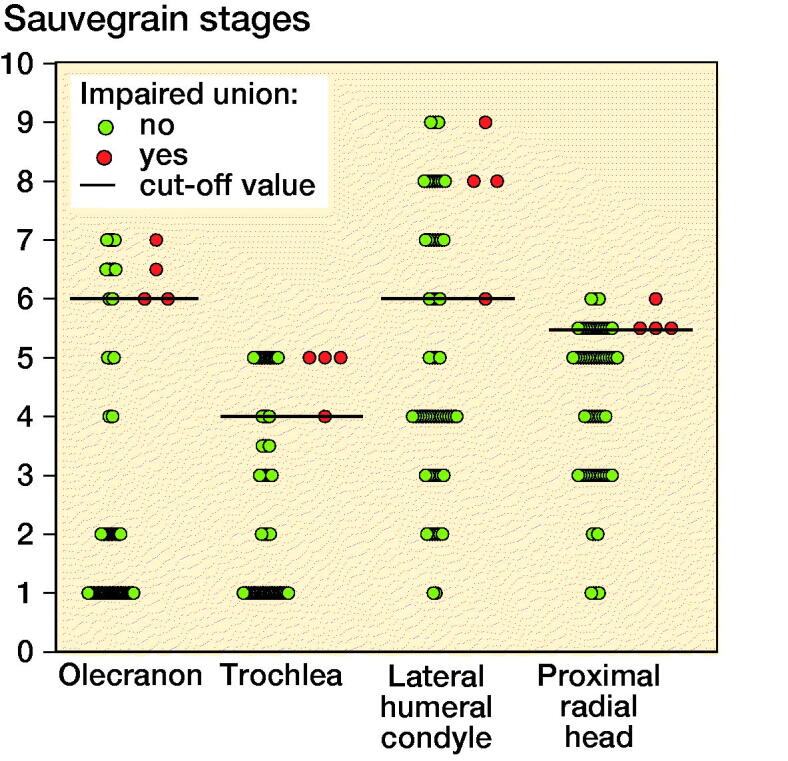
Optimal cutoff points (lines) of Sauvegrain and Dimйglio stages, according to area under receiver operating curve (ROC). Green dots are the fractures that united and red dots are the fractures that showed impaired union.

### Statistics

The receiver operating characteristic (ROC) curve was calculated to find the cutoff-points for Sauvegrain stages of 4 different ossification centers that identify impaired ossification. We also try to determine optimal cutoff value for calendar age; 9 years of age was indicative but statistical significance was not reached. Using the cutoff points of Sauvegrain classification for impaired union, diagnostic accuracy of the classification was evaluated by calculating sensitivity, specificity, positive predictive value, and negative predictive value with their 95% confidence intervals (CI). Furthermore, a standardized normal distribution (SND) exact test was used to compare the proportions of nonunion in classes defined by the cutoff values of olecranon stage and the indicative cutoff point for age (≥ 9 years) as well as sex. A logistic regression model was used to determine the risk of low-quality healing between the groups. The effect of higher chronological age on low-quality union was also tested by using age as a continuous variable in regression analysis. P < 0.05 was considered significant, requiring that all analyses were 2-sided. Statistics were calculated using StatsDirect Statistical Software (version 3.2.8, https://www.statsdirect.co.uk/, 2013) and the SPSS Statistical Package (version 26.0, IBM Corp, Armonk, NY, USA, 2019).

### Ethics, funding, and potential conflicts of interest

Following official instructions by the Ethical Board of Northern Finland Hospital District, Oulu, Finland, ethical board evaluation was waived and no ethics committee approval was needed. The approval by the local institution was obtained prior to study initiation. National research funding (VTR) was obtained for the study. Foundation of Pediatric Research have supported the study. This was a researcher-initiated study with no commercial conflict of interest. JJS was in receipt of a grant from the Pediatric Research Society, Alma and K.A. Snellman Foundation, Emil Aaltonen Foundation. NL and JJS are members of the European Paediatric Orthopaedic Society. The other authors declare no conflicts of interest. 

## Results (Figure 3)

### Olecranon ossification center

The optimal cutoff-point of the olecranon ossification center was 6, according to the ROC curve. All patients who suffered from impaired bone healing were identified with Sauvegrain stage 6–7 (sensitivity (Se) = 100%, CI 40–100%) (Table 1).

**Table 1.** Sensitivity, specificity, and predictive values of the elbow ossification centers, according to Sauvegrain and Diméglio development stage with particular cutoff points, in determining impaired union of forearm shaft fractures treated with elastic stable intramedullary nailing (ESIN)

	Area under ROC curve	Se (CI) **^a^**	Sp (CI)	PPV (CI)	NPV (CI) **^a^**
Olecranon	0.84	1 (0.40–1)	0.75 (0.64–0.85)	0.20 (0.06–0.44)	1 (0.93–1)
Trochlea	0.85	1 (0.40–1)	0.70 (0.57–0.80)	0.17 (0.05–0.37)	1 (0.92–1)
Lateral humeral condyle	0.84	1 (0.40–1)	0.61 (0.48–0.72)	0.13 (0.04–0.31)	1 (0.91–1)
Proximal radial head	0.87	0.71 (0.40–1)	0.73 (0.60–0.83)	0.18 (0.05–0.40)	1 (0.93–1)

ROC = receiver operating characteristic, Se = sensitivity, Sp = specificity, PPV = positive predictive value, NPV = negative predictive value, CI = 95% confidence interval.

a one-sided CI.

The development stage ≥ 6 of the olecranon in the primary radiographs was associated with impaired union in 4 out of 20 patients treated with ESIN, compared with none in olecranon stage 1–5 (CI of difference 8–42%). There was no difference in open fractures, higher displacement (> 5 mm vs. ≤ 5 mm) or open reduction (yes vs. no) between the patients with olecranon stage 6–7 vs. 1–5 (Table 2).

**Table 2.** Patients with higher vs. lower development stage of the elbow ossification centers were compared regarding severity of the fracture (higher displacement, open fracture) and open reduction

	Olecranon			Trochlea			Lateral humeral condyle			Proximal radial head		
	Stage	Stage		Stage	Stage		Stage	Stage		Stage	Stage	
	1–5	6–7		1–3.5	4–5		1–5	6–9		1–5	5.5–6	
	n = 50	n = 20	p-value	n = 46	n = 24	p-value	n = 40	n = 30	p-value	n = 48	n = 22	p-value
Impaired union, n	0	4	0.003	0	4	0.006	0	4	0.015	0	4	0.004
Difference, % (95% CI)	20 (8.0–42)			17 (6.6–36)			13 (3.4–30)			18 (7.3–39)		
Severity of the fracture												
Open fracture, n	5/50	5/20	0.08	4/45	6/24	0.05	3/39	7/30	0.05	4/47	6/22	0.04
Displaced > 5 mm, n	27/45	12/19	1.0	26/41	13/23	0.5	19/35	20/29	0.2	26/43	13/21	1.0
Open reduction, n	28/49	10/20	0.5	25/45	13/24	1.0	21/39	17/30	1.0	25/47	13/22	0.6
Age in years, mean (SD)	8.9 (2.3)	12 (2.3)	< 0.001	8.6 (2.2)	12 (2.1)	< 0.001	8.5 (2.2)	12 (2.1)	< 0.001	8.7 (2.3)	12 (2.0)	< 0.001

### Trochlea ossification center

With the ROC curve we found that the optimal cutoff point of the trochlear ossification center was 4. All fractures with impaired bone healing after ESIN were found to have trochlea stage 4–5 primarily. Sensitivity of this test was thus high (Se = 100%, CI 40–100%) in recognizing impaired union. Specificity (Sp) of test was 70% (CI 57–80%) (Table 1).

Trochlear ossification center ≥ 4 was associated with impaired bone healing in 4 out of 24 patients (CI of difference 7–36%), compared with none in trochlear stage 0–3.5. There seemed to be more open fractures, but no difference in displacement (> 5 mm vs. ≤ 5 mm) or open reduction (yes vs. no) between the patients with trochlea stage 4–5 vs. 1–3.5 was found (Table 2). 

### Lateral humeral condyle ossification center

Lateral humeral condyle ossification center stage 6 was found to be the optimal cutoff point according to ROC curve (Se = 100%, CI 40–100%). Specificity was 61% (CI 48–72%) (Table 1).

The development stage ≥ 6 of the lateral humeral condyle was associated with impaired union in 4 out of 30 patients (CI 3–30%) who were treated with ESIN. There seemed to be more open fractures among higher ossification stages. No difference was found in displacement (> 5 mm vs. ≤ 5 mm) or open reduction (yes vs. no) between the patients with lateral humeral stage 6–9 vs. stage 1–5 (Table 2).

### Proximal radial head ossification centers

The optimal cutoff point of Sauvegrain classification was 5.5 for the radial head, based on ROC curve. Sensitivity was 71% (CI 40–100%) and the specificity was 73% (CI 60–83%) (Table 1).

The development stage ≥ 5.5 of the proximal radial head was associated with impaired union in 4 out of 22 patients (CI 7.3–39%) who were treated with ESIN. There were more open fractures, but no difference in displacement (> 5 mm vs. ≤ 5 mm) or open reduction (yes vs. no) between the patients with proximal radial head stage 5.5–6 vs. 1–5 was seen (Table 2).

### Effect of age and sex

No statistically significant effect of inferior union was observed according to chronological age (p = 0.3) or between the age groups ≥ 9 years vs. < 9 years (p = 0.3). 1 out of 36 boys and 3 out of 34 girls presented low-quality fracture union (difference –6%, CI –21 to 7%). 

## Discussion

The main finding of this study was that the higher development stage of the olecranon ossification center, in particular, can be used to estimate the probability of impaired union of a forearm shaft fracture, when treated with ESIN. By using the method by Sauvegrain and Diméglio, we found that olecranon ossification center 6 or higher was associated with impaired ossification in 4 out of 20 patients. This means that a rectangular or fused olecranon ossification center rather than the more immature convex half-moon or lacking olecranon apophysis in the lateral radiographs may be associated with low-quality fracture healing, if ESIN is performed. In practical terms, the olecranon ossification center is easily seen on the conventional lateral view of the forearm radiograph and no extra radiographs are needed. 1 in 5 patients suffering from impaired ossification is a recognized number, given that most childhood fractures heal fast, meaning that our finding is clinically important.

The fundamental idea of the study method is based on the growing skeleton. During the growth period, there is cartilage between the metaphysis and epiphysis of the long bones and the calcification of the epiphyses is lacking. The timing of calcification of these secondary ossification centers in the period of growth after birth differs, meaning that calendar age is not accurate method in evaluating the physiological maturation of the skeleton in individual children. In contrary, bone age per se describes the stage of skeletal development in all people, irrespective of calendar age, sex, or ethnic group (Satoh 2015). There are several methods for assessing bone status in children, while the Risser bone maturity classification is still a reference method in many institutions (Thodberg et al. 2010). In scoliosis treatment, the method of Sauvegrain et al. (1962) for the assessment of skeletal age with use of radiographs of the elbow has been used (Charles et al. 2007). In general, complete ossification of the elbow secondary ossification centers correlates with full bone maturity. Modified by Diméglio et al (2005), Sauvegrain’s method is based on systematic and regular morphological development of the elbow apophyses during the accelerating growth phase in puberty (Diméglio et al. 2005, Charles et al. 2007). Assessment of these apophyses allows skeletal age to be evaluated accurately at 6-month intervals. The time of appearance of ossification centers around the elbow seems not to vary between sexes (Cheng et al. 1998). A rectangular or fused olecranon apophysis, instead of halfmoon circular, minor, or lacking apophysis refers to stage 6 or higher. In these circumstances, it was reasonable to hypothesize that skeletal maturation of the elbow area would aid in selecting the optimal treatment of forearm shaft fractures between child-type and adult-type procedures.

In addition to the olecranon ossification center, we found that the other 3 ossification centers, trochlea, lateral humeral condyle, and proximal radial head, were also associated with impaired union. This is reasonable, while all secondary ossification centers develop in a particular order and thus are related to bone age. However, although associated with impaired union, the other ossification centers had lower statistical variables when compared with the olecranon. From a clinical point of view, these other ossification centers may support the clinician as well, when he/she is considering the maturation stage of the patient’s skeleton. Our finding regarding other ossification centers is different from that of the report by Morrison et al. (2020), which is the only previous study of the issue. They reported on only the olecranon apophysis and its association with inferior results of ESIN in childhood forearm shaft fractures. In their study, olecranon stage > 3 (on the scale 1–7) was associated with increased complications in bone healing. In our study, the optimal cutoff point was higher: no patient presented disturbed healing if the olecranon development status was between 1 and 5. However, there was a difference in determining low-quality fracture healing between the studies: Morrison et al. used 6 months of impaired healing as nonunion, while the respective time was 12 months in our study.

The study question of whether to use ESIN or other methods in older children with unstable forearm shaft fractures is clinically essential, bearing in mind that forearm shaft fractures are usual in that age group and their incidence is still increasing (Mäyränpää et al. 2010). There are many technical possibilities in performing operative stabilization, including open reduction and internal plate and screw fixation (ORIF) and intramedullary nailing with flexible nails (elastic stable intramedullary nailing, ESIN) (Bochang et al. 2005, Fernandez et al. 2010). Several implant materials have been used in surgical fixation of forearm shaft fractures, such as stainless steel, titanium alloy, and biodegradable composites (Van der Reis et al. 1998, Colaris et al. 2013, Korhonen et al. 2018). ESIN is currently the preferred method of surgical fixation in children, compared with ORIF, due to several advantages such as better cosmesis, decreased operative time, early return to activities, intact fracture hematoma, and good union rate (Lascombes et al. 1990, Schmittenbecher 2005). However, from the clinical point of view, there is still some controversy in the indications as to whether to use ESIN or plating in older children and adolescents, regardless of encouraging evidence in younger children (Ortega et al. 1996, Baldwin et al. 2014). The displacing muscle forces are greater in adolescents, the length of the shaft is higher resulting in greater torque at the fracture, the remodeling capacity is lower, and bone turnover rate is slower in adolescents, compared with younger children (Ortega et al. 1996, Sinikumpu and Serlo 2015). Thus, using the optimal method of treatment is particularly important in forearm midshaft fractures; as opposed to many other pediatric fractures, there is a risk of impaired healing in these bones, especially the ulna. Delayed union is reported to occur in 7% of patients and the risk of nonunion is 1–3% (Mehlman and Wall 2006, Schmittenbecher et al. 2008, Sinikumpu et al. 2013). The ulna is a subcutaneous bone and more prone to nonunion than the radius (Fernandez et al. 2009). Furthermore, there is limited remodeling in the forearm diaphysis, which is far from the metabolically active growth plates. Malunited fractures tend to be associated with decreased forearm rotation, resulting in a more aggressive approach with surgical stabilization often being required (Kutsikovich et al. 2018).

The strength of our study is that all children treated operatively for forearm shaft fractures during the recruitment period were enrolled. The patients were treated according to best available practice. Their treatment was based on the authentic decision of the treating surgeon. Another strength is that the patients with higher maturation stage of the ossification centers were similar regarding fracture severity and the need for open reduction, when compared with the more immature patients. This supports the hypothesis that no confusing fracture- or surgery-related factors affected the results, but impaired union was associated with a higher stage of elbow ossification centers. Follow-up was based on normal practice in the institution and there was no loss of follow-up. Another fact that emphasizes the value of this study is that there is no evidence-based level I or II data supporting either ORIF or ESIN in children’s forearm fractures (Abraham et al. 2011, Baldwin et al. 2014). The chronological calendar age cannot be used as a determinant for any surgical procedure in forearm shaft fractures. In our study, there was no statistically significant classifying value for optimal cutoff age point that would predict low-quality fracture union. Higher calendar age had no statistically significant effect on impaired fracture healing in the patients in this study.

A limitation is that the material was collected retrospectively and no causality but only statistical association could be evaluated. In addition, the study model was not validated in any external dataset. There were several patients who were treated by other surgical methods, such as biodegradable implants or Kirschner wires, and thus were excluded. Further, no data of long-term outcomes was available. High sensitivity of the Sauvegrain method of olecranon classification means that stages 1–5 (negative result test) ruled out impaired union but a wide (97.5%) confidence interval and in particular its lower bound (40%) indicate that 100% sensitivity of the test overestimates the result. This means that not all patients with an ossification center higher than the determined optimal cutoff point suffered from impaired healing. The positive predictive value (PPV = 20%, CI 6–44%) means that the number of false-positive test results was high (80%). For these false positives, ESIN would still be the appropriate method of treatment, resulting in good bone healing, which needs to be emphasized. From a clinical point of view, a minority of the patients with higher Sauvegrain stage of the olecranon suffered from impaired union. This highlights that ESIN is in general a superior method in treating pediatric forearm shaft fractures. The overall risk of disturbed healing of forearm shaft fractures treated with ESIN is in general low, and selection of the surgical procedure needs to be decided individually for every patient. However, a 20% of risk of impaired bone healing is still a high rate of impaired recovery in the growing skeleton and the method we describe may aid in improving the treatment of these particular patients: as a straightforward way to assess the bone maturity of each patient, this method could give additional information for a surgeon treating a child with a forearm shaft fracture. As another limitation, the number of patients with impaired bone healing was low, which justifies further studies in greater study settings. Larger studies are important to further analyze the effect of gender. However, in the authors’ understanding, the reported method could be feasible for both sexes, given that the bone maturation process itself is not dependent on gender, and the development of secondary ossification centers around the elbow does not differ between the sexes (Cheng et al. 1998, Satoh. 2015).

In conclusion, in this study we found that the rectangular shape of olecranon maturation stage 6 or higher, in particular, seen on the lateral view of conventional forearm radiographs, can be used when considering the different treatment methods for older children and teenagers with forearm shaft fractures.

## References

[CIT0001] Abraham A, Kumar S, Chaudhry S, Ibrahim T. Surgical interventions for diaphyseal fractures of the radius and ulna in children. Cochrane Database Syst Rev 2011; (11): CD007907. 22071838 10.1002/14651858.CD007907.pub2

[CIT0002] Alrashedan B S, Jawadi A H, Alsayegh S O, Alshugair I F, Alblaihi M, Jawadi T A, Hassan A A, Alnasser A M, Aldosari N B, Aldakhail M A. Outcome of diaphyseal pediatric forearm fractures following non-surgical treatment in a level I trauma center. Int J Health Sci 2018; 12: 60. PMC6124831 30202409

[CIT0003] Asadollahi S, Pourali M, Heidari K. Predictive factors for re-displacement in diaphyseal forearm fractures in children-role of radiographic indices. Acta Orthop 2017; 88(1): 101–8. 27841692 10.1080/17453674.2016.1255784 PMC5251255

[CIT0004] Baldwin K, Morrison 3rd M J, Tomlinson L A, Ramirez R, Flynn J M. Both bone forearm fractures in children and adolescents, which fixation strategy is superior—plates or nails? A systematic review and meta-analysis of observational studies. J Orthop Trauma 2014; 28(1): e8–e14. 23542745 10.1097/BOT.0b013e31829203ea

[CIT0005] Bhandari M, Guyatt G H, Swiontkowski M F, Tornetta 3rd P, Sprague S, Schemitsch E H. A lack of consensus in the assessment of fracture healing among orthopaedic surgeons. J Orthop Trauma 2002; 16(8): 562–6. 12352564 10.1097/00005131-200209000-00004

[CIT0006] Bochang C, Jie Y, Zhigang W, Weigl D, Bar-On E, Katz K. Immobilisation of forearm fractures in children: extended versus flexed elbow. J Bone Joint Surg Br 2005; 87(7): 994–6. 15972919 10.1302/0301-620X.87B7.15774

[CIT0007] Charles Y P, Diméglio A, Canavese F, Daures J. Skeletal age assessment from the olecranon for idiopathic scoliosis at Risser grade 0. J Bone Joint Surg Am 2007; 89(12): 2737–44. 18056507 10.2106/JBJS.G.00124

[CIT0008] Cheng J C, Wing-Man K, Shen W Y, Yurianto H, Xia G, Lau J T, Cheung A Y. A new look at the sequential development of elbow-ossification centers in children. J Pediatr Orthop 1998; 18(2): 161–7. 9531396

[CIT0009] Colaris J W, Allema J H, Reijman M, Biter L U, De Vries M R, Van De Ven, C P, Bloem R M, Verhaar J A N. Risk factors for the displacement of fractures of both bones of the forearm in children. Bone Joint J 2013; 95-B(5) (5): 689–93. 23632683 10.1302/0301-620X.95B5.31214

[CIT0010] Diméglio A, Charles Y P, Daures J, De Rosa V, Kaboré B. Accuracy of the Sauvegrain method in determining skeletal age during puberty. J Bone Joint Surg Am 2005; 87(8): 1689–96. 16085606 10.2106/JBJS.D.02418

[CIT0011] Fernandez F, Eberhardt O, Langendörfer M, Wirth T. Nonunion of forearm shaft fractures in children after intramedullary nailing. J Pediatr Orthop B 2009; 18(6): 289–95. 19623087 10.1097/BPB.0b013e32832f5b20

[CIT0012] Fernandez F, Langendörfer M, Wirth T, Eberhardt O. Failures and complications in intramedullary nailing of children’s forearm fractures. J Child Orthop 2010; 4(2): 159–67. 21455473 10.1007/s11832-010-0245-y PMC2839862

[CIT0013] Franklin C C, Robinson J, Noonan K, Flynn J M. Evidence-based medicine: management of pediatric forearm fractures. J Pediatr Orthop 2012; 32: 131. 10.1097/BPO.0b013e318259543b 22890452

[CIT0014] Herman M J, Marshall S T. Forearm fractures in children and adolescents: a practical approach. Hand Clin 2006; 22(1): 55–67. 16504778 10.1016/j.hcl.2005.10.003

[CIT0015] Joeris A, Lutz N, Blumenthal A, Slongo T, Audigé L. The AO pediatric comprehensive classification of long bone fractures (PCCF) part I: location and morphology of 2,292 upper extremity fractures in children and adolescents. Acta Orthop 2017; 88(2): 129–32. 27882811 10.1080/17453674.2016.1258533 PMC5385105

[CIT0016] Korhonen L, Perhomaa M, Kyrö A, Pokka T, Serlo W, Merikanto J, Sinikumpu J. Intramedullary nailing of forearm shaft fractures by biodegradable compared with titanium nails: results of a prospective randomized trial in children with at least two years of follow-up. Biomaterials 2018; 185: 383–92. 30292588 10.1016/j.biomaterials.2018.09.011

[CIT0017] Kutsikovich J I, Hopkins C M, Gannon 3rd E W, Beaty J H, Warner W C, Sawyer J R, Spence D D, Kelly D M. Factors that predict instability in pediatric diaphyseal both-bone forearm fractures. J Pediatr Orthop B 2018; 27(4): 304–8. 28777160 10.1097/BPB.0000000000000480

[CIT0018] Lascombes P, Prevot J, Ligier J N, Metaizeau J P, Poncelet T. Elastic stable intramedullary nailing in forearm shaft fractures in children: 85 cases. J Pediatr Orthop 1990; 10(2): 167–71. 2312694

[CIT0019] Mäyränpää M K, Mäkitie O, Kallio P E. Decreasing incidence and changing pattern of childhood fractures: a population–based study. J Bone Miner Res 2010; 25(12): 2752–9. 20564246 10.1002/jbmr.155

[CIT0020] Mehlman C T, Wall E J.Injuries to the shafts of the radius and ulna. In: Beaty J H, Kasser JR, editors. Rockwood and Wilkins’ fractures in children. VI ed. Philadelphia: Lippincott Williams & Wilkins 2006. p. 399–441.

[CIT0021] Morrison 3rd M J, Speirs J N, Chicorelli A M, Garner M, Flynn J J M, Herman M J. Intramedullary fixation of both bone forearm fractures in children and adolescents: healing correlates with development of the olecranon apophysis. J Pediatr Orthop 2020; 40(3): e198–e202. 31219914 10.1097/BPO.0000000000001419

[CIT0022] Ortega R, Loder R T, Louis D S. Open reduction and internal fixation of forearm fractures in children. J Pediatr Orthop 1996; 16(5): 651–4. 8865053 10.1097/00004694-199609000-00019

[CIT0023] Rehman S, Sokunbi G. Intramedullary fixation of forearm fractures. Hand Clin 2010; 26(3): 391–401. 20670804 10.1016/j.hcl.2010.04.002

[CIT0024] Satoh M. Bone age: assessment methods and clinical applications. Clin Pediatr Endocrinol 2015; 24(4): 143–52. 26568655 10.1297/cpe.24.143 PMC4628949

[CIT0025] Sauvegrain J, Nahum H, Bronstein H. Study of bone maturation of the elbow. Ann Radiol (Paris) 1962; 5: 542–50. 13986863

[CIT0026] Schmittenbecher P P. State-of-the-art treatment of forearm shaft fractures. Injury 2005; 36(1): 25. 10.1016/j.injury.2004.12.010 15652933

[CIT0027] Schmittenbecher P, Fitze G, Gödeke J, Kraus R, Schneidmüller D. Delayed healing of forearm shaft fractures in children after intramedullary nailing. J Pediatr Orthop 2008; 28(3): 303–6. 18362794 10.1097/BPO.0b013e3181684cd6

[CIT0028] Sinikumpu J, Serlo W. The shaft fractures of the radius and ulna in children: current concepts. J Pediatr Orthop B 2015; 24(3): 200–6. 25714940 10.1097/BPB.0000000000000162

[CIT0029] Sinikumpu J, Lautamo A, Pokka T, Serlo W. The increasing incidence of paediatric diaphyseal both-bone forearm fractures and their internal fixation during the last decade. Injury 2012; 43(3): 362–6. 22154046 10.1016/j.injury.2011.11.006

[CIT0030] Sinikumpu J, Pokka T, Willy S. The changing pattern of pediatric both-bone forearm shaft fractures among 86,000 children from 1997 to 2009. Eur J Pediatr Surg 2013; 23(04): 289–96. 23444075 10.1055/s-0032-1333116

[CIT0031] Slongo T F, Audigé L, AO Pediatric Classification Group. Fracture and dislocation classification compendium for children: the AO pediatric comprehensive classification of long bone fractures (PCCF). J Orthop Trauma 2007; 21(10 Suppl.): 135. 10.1097/00005131-200711101-00020 18277238

[CIT0032] Thodberg H H, van Rijn R R, Tanaka T, Martin D D, Kreiborg S. A paediatric bone index derived by automated radiogrammetry. Osteoporos Int 2010; 21(8): 1391–400. 19937229 10.1007/s00198-009-1085-9 PMC2895878

[CIT0033] Van der Reis W, Otsuka N Y, Moroz P, Mah J. Intramedullary nailing versus plate fixation for unstable forearm fractures in children. J Pediatr Orthop 1998; 18: 9–13. 9449094

[CIT0034] Wall L B. Staying out of trouble performing intramedullary nailing of forearm fractures. J Pediatr Orthop 2016; 36: 71. 10.1097/BPO.0000000000000760 27078233

[CIT0035] Zionts L E, Zalavras C G, Gerhardt M B. Closed treatment of displaced diaphyseal both-bone forearm fractures in older children and adolescents. J Pediatr Orthop 2005; 25(4): 507–12. 15958905 10.1097/01.bpo.0000158005.53671.c4

